# Senolytic Treatment Reduces Acute and Chronic Lung Inflammation in an Aged Mouse Model of Influenza

**DOI:** 10.1111/acel.70480

**Published:** 2026-04-08

**Authors:** Lou Delval, Marybeth Creskey, Clara Valentin, Clément Bordas, Stefano Raviola, Larissa Lipskaia, Séverine Heumel, Lucie Deruyter, Valentin Sencio, Isabelle Wolowczuk, David Bernard, Serge Adnot, Philippe Gosset, Xu Zhang, François Trottein

**Affiliations:** ^1^ CNRS, INSERM, CHU Lille, Institut Pasteur de Lille, U1019—UMR 9017—CIIL—Center for Infection and Immunity of Lille Univ. Lille Lille France; ^2^ Regulatory Research Division, Biologic and Radiopharmaceutical Drugs Directorate Health Products and Food Branch, Health Canada Ottawa, Canada University Ottawa Canada; ^3^ INSERM U955, Institut Mondor de Recherche Biomédicale (IMRB), FHU SENEC University Paris‐Est Créteil Créteil France; ^4^ Département de Physiologie‐Explorations Fonctionnelles and FHU SENEC Hôpital Henri Mondor, AP‐HP Créteil France; ^5^ Equipe Labellisée la Ligue Contre le Cancer, Centre de Recherche en Cancérologie de Lyon, Inserm U1052, CNRS UMR 5286, Centre Léon Bérard Université de Lyon Lyon France; ^6^ School of Pharmaceutical Sciences, Faculty of Medicine University of Ottawa Ottawa Canada

**Keywords:** acute response, advanced age, cellular senescence, chronic damage, dysbiosis, inflammation, influenza, senolysis

## Abstract

Influenza A virus continues to pose a significant global health burden, with older individuals experiencing disproportionate morbidity and mortality. Although aging is associated with the accumulation of senescent cells, the extent to which cellular senescence contributes to influenza severity remains poorly understood. The aim of this study was to evaluate the therapeutic potential of the senolytic drug ABT‐263, a B cell lymphoma‐2 inhibitor, in mitigating both acute and chronic damage in an aged mouse model of influenza. Early administration of ABT‐263, beginning one day prior to infection, did not prevent body weight loss, reduce pulmonary viral load, or improve clinical scores in aged mice. However, ABT‐263 treatment significantly reduced lung inflammation in aged mice, coinciding with changes in the expression of senescence‐associated markers. ABT‐263 also reduced intestinal inflammation and mitigated virus‐induced gut dysbiosis, a known contributor to disease severity and secondary outcomes. Although the treatment lowered antigen‐specific CD8^+^ T cell responses, it did not affect influenza‐specific antibody production nor pulmonary defense against reinfection. Notably, ABT‐263 reduced long‐term pulmonary sequelae including edema, type II hyperplasia, emphysema and epithelial damage. Overall, ABT‐263 therapy partially mitigates influenza severity in aged mice, primarily through dampening acute and chronic inflammation. Most of these effects were age‐dependent, suggesting a role for pre‐existing senescent cells.

## Graphical abstract

Removing age‐related senescent cells before influenza infection in aged mice did not reduce viral replication but lessened lung pathology and gut dysbiosis, which contribute to disease severity. Treatment also mitigated long sequelae in lungs. Effects were mostly age‐dependent, implicating pre‐existing senescent cells in disease severity.

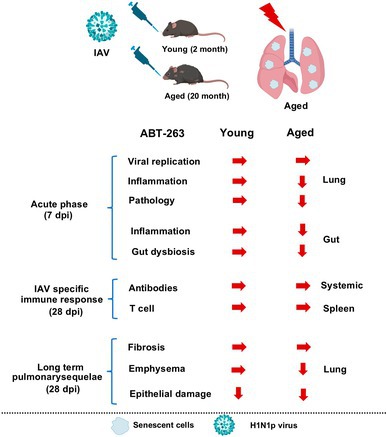

**Abbreviations**
Bcl‐2: B cell lymphoma‐2
dpi: day post‐infection
IAV: Influenza A virus
SASP: senescence‐associated secretory phenotype

## 1 Introduction

Globally, influenza A virus (IAV) affects hundreds of millions of people annually and a significant proportion die from pneumonia or extra‐pulmonary complications (Iuliano et al. 2018; Peteranderl et al. 2016; Thompson et al. 2004). It is estimated that seasonal flu kills between 200,000 and 600,000 persons per year and that millions experience long‐term complications (Xie et al. 2024). In addition to the recurrent medical and socio‐economic impacts caused by seasonal influenza each year, the emergence of a reassorted human‐pathogenic IAV strain, which occurs every 20–30 years, can be devastating (Taubenberger and Morens 2006; Peteranderl et al. 2016). Older adults, particularly those aged 65 years and above, are especially susceptible to influenza (Keilich et al. 2019). Several factors contribute to enhanced susceptibility and prolonged outcomes including the diminished pulmonary immune defenses, the alteration of lung functions (e.g., mucociliary clearance, barrier), and the lower capacity to repair tissue damage (Boe et al. 2017; Schneider et al. 2021; Kasmani et al. 2023). How these changes enhance susceptibility to infections and influence disease progression and clinical outcomes is the subject of intensive research. Indeed, with the global population aging, there is an urgent need for preventive and therapeutic strategies to improve influenza outcomes in this highly susceptible population. In the current study, we investigate the potential role of cellular senescence, a hallmark of aging (López‐Otín et al. 2023), in experimental influenza.

Cellular senescence arises following stresses such as oxidative stress, DNA‐damaging agents, inflammation, infection, and telomere shortening (Muñoz‐Espín and Serrano 2014; Di Micco et al. 2021). Cellular senescence is characterized by several features, such as the loss of a cell's ability to divide and replicate, along with an increased resistance to apoptosis (Muñoz‐Espín and Serrano 2014). The latter is due at least in part to members of the B cell lymphoma‐2 family of proteins (Bcl2, Bcl‐xL, and Bcl‐w) (Gorgoulis et al. 2019). Although senescent cells no longer proliferate, they remain transcriptionally and metabolically active, releasing a variety of pro‐inflammatory and immunomodulatory cytokines, growth factors, and proteolytic enzymes. This collection of secreted factors, known as the senescence‐associated secretory phenotype (SASP), exerts significant effects on neighboring cells and tissues (Birch and Gil 2020; Gorgoulis et al. 2019). Cellular senescence serves multiple physiological functions throughout life. Initially, it plays a crucial role in the formation of tissues and organs during embryonic development. Later in life, senescent cells can be beneficial in certain contexts (e.g., tumor suppression, tissue repair), and their presence is tightly regulated by the immune system (Muñoz‐Espín and Serrano 2014). In contrast, the aberrant accumulation of senescent cells in older adults—partly due to chronic stress, inflammaging, gut dysbiosis, and/or impaired immunity—has detrimental consequences. Experimental and clinical evidence show that the accrual of senescent cells participates in many age‐related declines and diseases of aging (Kirkland and Tchkonia 2017; Baker et al. 2011; Gorgoulis et al. 2019; Childs et al. 2017). In the lungs, age‐related cellular senescence contributes to the altered pulmonary functions (Parikh et al. 2019). Studies have shown that the genetic or pharmacological removal of age‐related senescent cells improves disease outcomes and extends lifespan (Baker et al. 2016; Chang et al. 2016; Kirkland and Tchkonia 2017; Tchkonia et al. 2013).

The potential role of age‐related, senescent cells during viral infections has recently been investigated (Torrance et al. 2024). Using a model of systemic coronavirus infection, Camell and colleagues demonstrated that the removal of senescent cells improves disease outcomes in aged mice (Camell et al. 2021). In the same line, we found that depletion of age‐related senescent cells reduces the severity of coronavirus disease 19 (COVID‐19) in aged hamsters (Delval et al. 2023). Regarding influenza, the situation is less clear and data are controversial. Some studies have reported a beneficial effect of senescent cell depletion on influenza outcomes in aged mice (Lv et al. 2022; Chen et al. 2022; Jiang et al. 2025; Torrance et al. 2024), whereas others have found that senolytic agents have no significant impact in this context (Torrance et al. 2023; Luna et al. 2025). These contradictory findings warrant further investigation. In addition, the long‐term pathological effects of senolytic treatment, as well as potential extra‐pulmonary disorders, remain to be elucidated. Our findings suggest that pharmacological removal of senescent cells can partially reduce influenza severity in aged mice, with the greatest impact on both acute and chronic inflammatory responses. These results were age‐dependent (with the exception of epithelial recovery), suggesting that pre‐existing senescent cells contribute to disease severity.

## 2 Results

### 2.1 ABT‐263 Treatment in Aged Mice Has No Effect on Viral Load nor Clinical Outcomes but Reduces Pulmonary Inflammation

**FIGURE 1:**
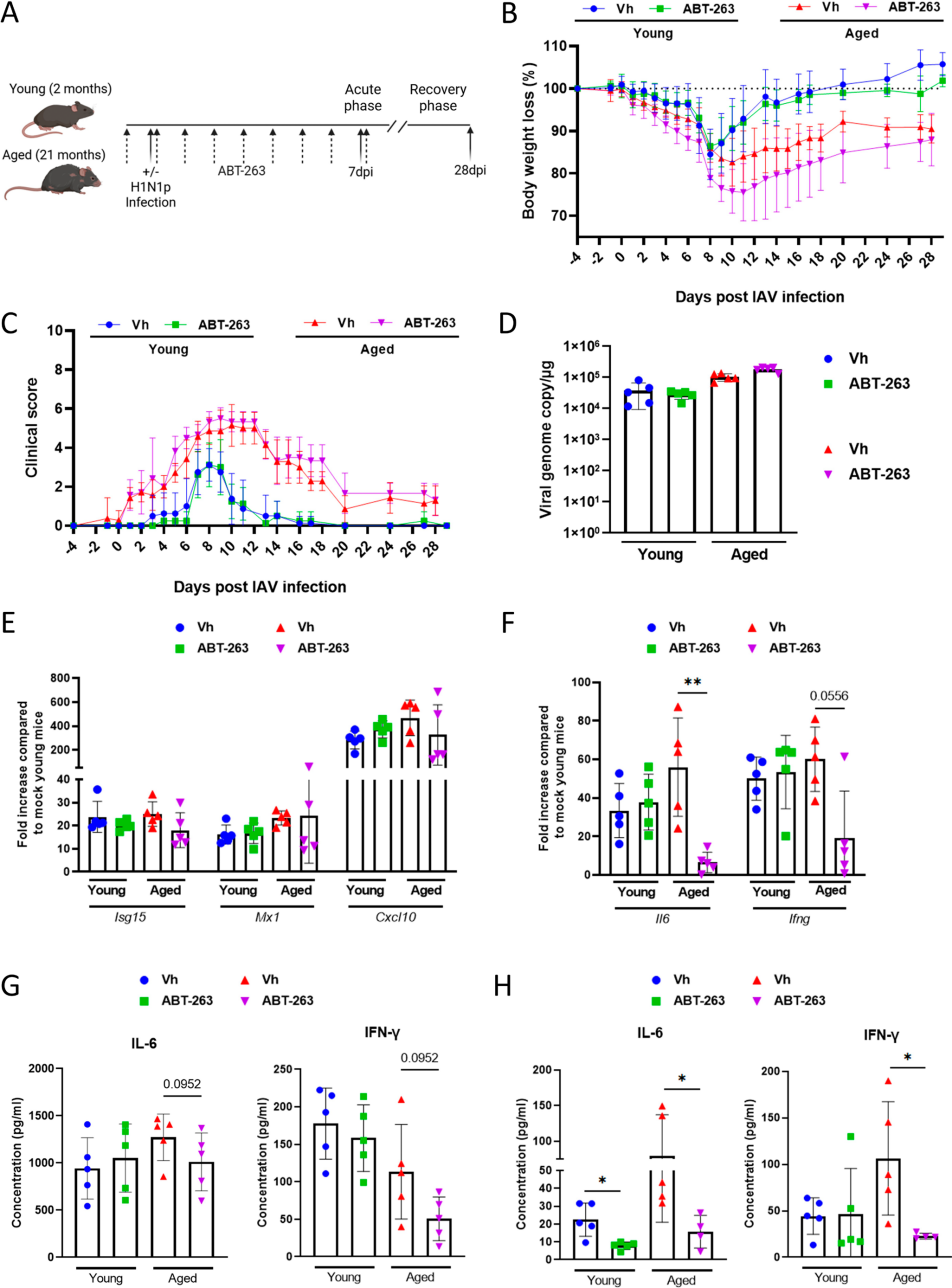
Effect of ABT‐263 treatment on the acute phase response of influenza. (A) Young and aged male C57BL/6 mice were treated with ABT‐263 starting one day before IAV infection and then daily until 7 dpi. Mice were sacrificed at 7 or 28 dpi. (B, C) Measurement of body weight loss and regain and clinical scores during the course of infection. (D) Quantification of viral load in the whole lung using specific TaqMan RT‐qPCR (7 dpi). Data are expressed as genome copy number (M1 protein)/μg RNA. (E, F) mRNA copy numbers were quantified by quantitative RT‐PCR. The data are expressed as the mean of change relative to average gene expression in non‐infected young animals. (G, H) Proinflammatory cytokines in lungs (G) and plasma (H) were quantified by ELISA. (A–H). Errors indicate mean ± SD (*n* = 5). One representative experiment out of two performed are depicted. Significant differences were determined using the Kruskal–Wallis test (*p < 0.05, **p < 0.01).

To study the potential involvement of age‐associated senescent cells in influenza pathogenesis, we use ABT‐263, also known as navitoclax. This senolytic drug targets the anti‐apoptotic Bcl‐2 family proteins, breaking the lock allowing senescent cells to resist to apoptosis (Zhu et al. 2016; Chang et al. 2016). Young adult (2 months old) and aged (21 months old) mice were orally treated with ABT‐263 (or with the vehicle) starting one day before infection with the mouse‐adapted IAV strain H1N1 A/California/04/2009 (pdm09). Treatment continued daily until Day 7 post‐infection (dpi), corresponding to the peak of the acute phase (Figure 1A). Our objective was to deplete pre‐existing, age‐related senescent cells prior to influenza and to eliminate senescent cells early induced by the infection. This protocol has been successfully implemented in a COVID‐19‐like disease model in aged hamsters (Delval et al. 2023). As expected, aged animals experienced more severe symptoms compared to young animals. Vehicle‐treated aged mice lost more body weight (peak at 10 dpi) relative to young counterparts (peak at 8 dpi) and failed to recover their initial body weight at 28 dpi (−9%) (Figures 1B and S1A). In contrast, young mice regained their initial body weight by 18 dpi. While ABT‐263 had no effect on body weight loss or recovery in young animals, it tended to exacerbate body weight loss in aged mice (not significant). Notably, ABT‐263‐treated aged mice recovered body weight at 28 dpi to a similar extent as vehicle‐treated aged mice. Clinical scores (Table 1) were more severe and prolonged in vehicle‐treated aged animals compared to younger counterparts (Figures 1C and S1B). ABT‐263 treatment had no effect on clinical scores, regardless of the age group.

**TABLE 1:** Clinical scoring system to assess the severity of disease in an experimental model of influenza.

	0	1	2	3
Activity	Normal	Alert/slow movement	Lethargic/trembling	Inactive unless physically stimulated
Posture	Normal	Hunched back	Wasted/emaciated	Crouched, head down
Fur	Normal	Piloerection	Rough coat	Very ruffled/unkempt/dirty
Eyes/Nose	Normal	Half‐closed/squinting	Squinting/discharge	Closed/discharge
Weight	Within ±5% of baseline	5%–10% weight loss	10%–19% weight loss	> 20% weight loss

*Note:* For each parameter: minimum score of 0 and maximum score of 3.

We then analyzed the impact of ABT‐263 treatment on viral load in the lungs at 7 dpi. Previous studies have shown that viral replication—including that of IAV—can be enhanced in senescent cells (Schulz et al. 2023; Urata et al. 2022; Hsieh et al. 2020; Malikova et al. 2021). However, other reports suggest that senescence‐associated factors, such as p21, may instead inhibit viral replication (AbuBakar et al. 2014; Baz‐Martínez et al. 2016; Ma et al. 2022). As depicted in Figure 1D, ABT‐263 treatment had no significant effect on pulmonary viral load in either young or aged mice. At 7 dpi, the expression of interferon‐stimulated genes (ISGs) generally mirrors the viral burden. Quantitative RT‐PCR analysis revealed that ABT‐263 has no significant impact on the expression of ISG transcripts including Isg15, Mx1, and Cxcl10 in either age group (Figure 1E). In contrast, ABT‐263 treatment in aged—but not young—mice reduced the transcript expression levels of some, but not all, SASP‐related compounds such as interleukin (IL)‐6 and IFN‐γ (Figure 1F). In line, the pulmonary (*p* = 0.0952) and blood protein levels of IL‐6 and IFN‐γ were reduced in ABT‐263‐treated aged animals (Figure 1G,H). The age‐specific effect of ABT‐263 in modulating the inflammatory response during infection in aged mice suggests a contributory role for pre‐existing senescent cells.

### 2.2 ABT‐263 Treatment Ameliorates Acute Pulmonary Influenza Disease in Aged Mice

**FIGURE 2:**
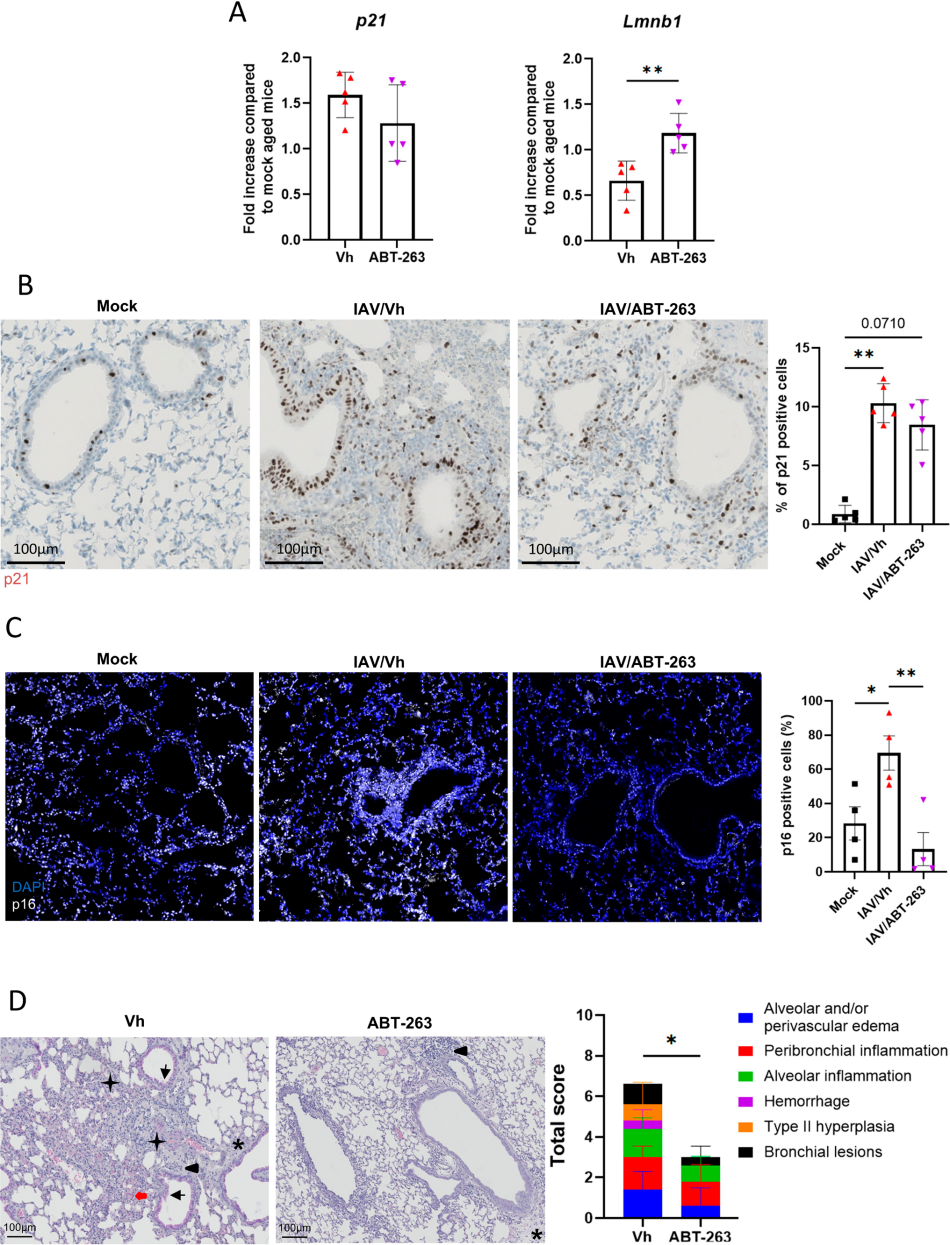
Effect of ABT‐263 treatment on lung inflammation and on the expression of senescent markers. Infected mice were sacrificed at 7 dpi. (A) Expression of p21 and Lmnb1 transcripts in the lung of vehicle‐treated and ABT‐263‐treated aged mice (7 dpi) as determined by RT‐qPCR. (B) Left panels, Lungs from aged mice were stained with anti‐p21 antibodies (7 dpi). Scale bars, 100 μm. Right panel, The percentages of p21‐positive cells are indicated (*n* = 5). (C) Left panels, Lungs from aged mice were stained with anti‐p16 antibodies (7 dpi). Scale bars, 100 μm. Right panel, The percentages of p16‐positive cells are indicated (*n* = 4). (D) Lungs were stained with H&E. Representative photomicrographs are shown. *, Alveolar and/or perivascular edema; ▲, Peribronchial inflammation; →, Hemorrhage; ★, Type II pneumocyte hyperplasia; ←, Bronchial lesions. Scale bars, 100 μm. Left panel, The mean sum of the subscores is shown. (A–D) Errors indicate mean ± SD (*n* = 5). One representative experiment out of two performed are depicted. Significant differences were determined using the Mann Whitney *U* test (A–C) or the Kruskal–Wallis test (D) (*p < 0.05, **p < 0.01).

We then investigated whether the anti‐inflammatory effects of ABT‐263 in aged mice were associated with a reduced burden of senescent cells in the lungs. To this end, the expression of the early senescent marker p21 was measured by quantitative RT‐PCR in whole lung tissue. Compared to vehicle‐treated controls, ABT‐263 treatment in aged mice tended to lower the transcript expression of p21 (Figure 2A, left panel). Loss of nuclear lamin b1 is also considered as a biomarker of senescence (Freund et al. 2012). In line with the previous finding, the transcript levels of lamin b1 were significantly enhanced in ABT‐263‐treated aged mice (Figure 2A, right panel). The effect of ABT‐263 on *p21* and *Lmnb1* gene expression were age‐specific (Figure S2A). We then analyzed p21 protein expression in lungs. Immunohistochemistry on lung sections indicated that p21 expression is dramatically enhanced in aged and young at 7 dpi (Figures 2B and S2B). p21 principally locates in bronchial epithelium and in inflammatory infiltrates. ABT‐263 tended to lower p21 protein expression in aged lungs (Figure 2B). Relative to mock‐infected mice, immunofluorescence analysis revealed that the expression of the senescent marker p16 was increased in aged mice at 7 dpi. Treatment with ABT‐263 significantly reduced its expression (Figure 2C).

We then analyzed the impact of ABT‐263 on lung pathology at 7 dpi. To this end, lung sections were stained with hematoxylin & eosin (H&E) to assess histopathological changes. Following infection, both young and aged mice exhibited bronchoepithelial hyperplasia, bronchointerstitial pneumonia, and mixed inflammatory cell infiltrates in perivascular and intra‐alveolar areas (Figures 2D and S2C, left panels). Semiquantitative scoring revealed that aging increased lung inflammation and tissue damage (more bronchial, alveolar and perivascular lesions than young mice) (Figures 2D and S2C, right panel). Interestingly, ABT‐263 treatment significantly diminished pathological multiparametric scores in aged animals. In particular, ABT‐263 abrogated, albeit not significantly, bronchial lesions and type II pneumocyte hyperplasia as well as alveolar and perivascular edemas (Figure 2D). In line with our prior finding (Lipskaia et al. 2025), ABT‐263 treatment had no effect on pulmonary pathology at 7 dpi in young animals (Figure S2C). In summary, ABT‐263 treatment in aged mice reduced lung inflammation and histopathological damage during IAV infection, effects that were associated with reduced markers of cellular senescence.

### 2.3 Gut Disorders Are Reduced in ABT‐263‐Treated Aged Mice

**FIGURE 3:**
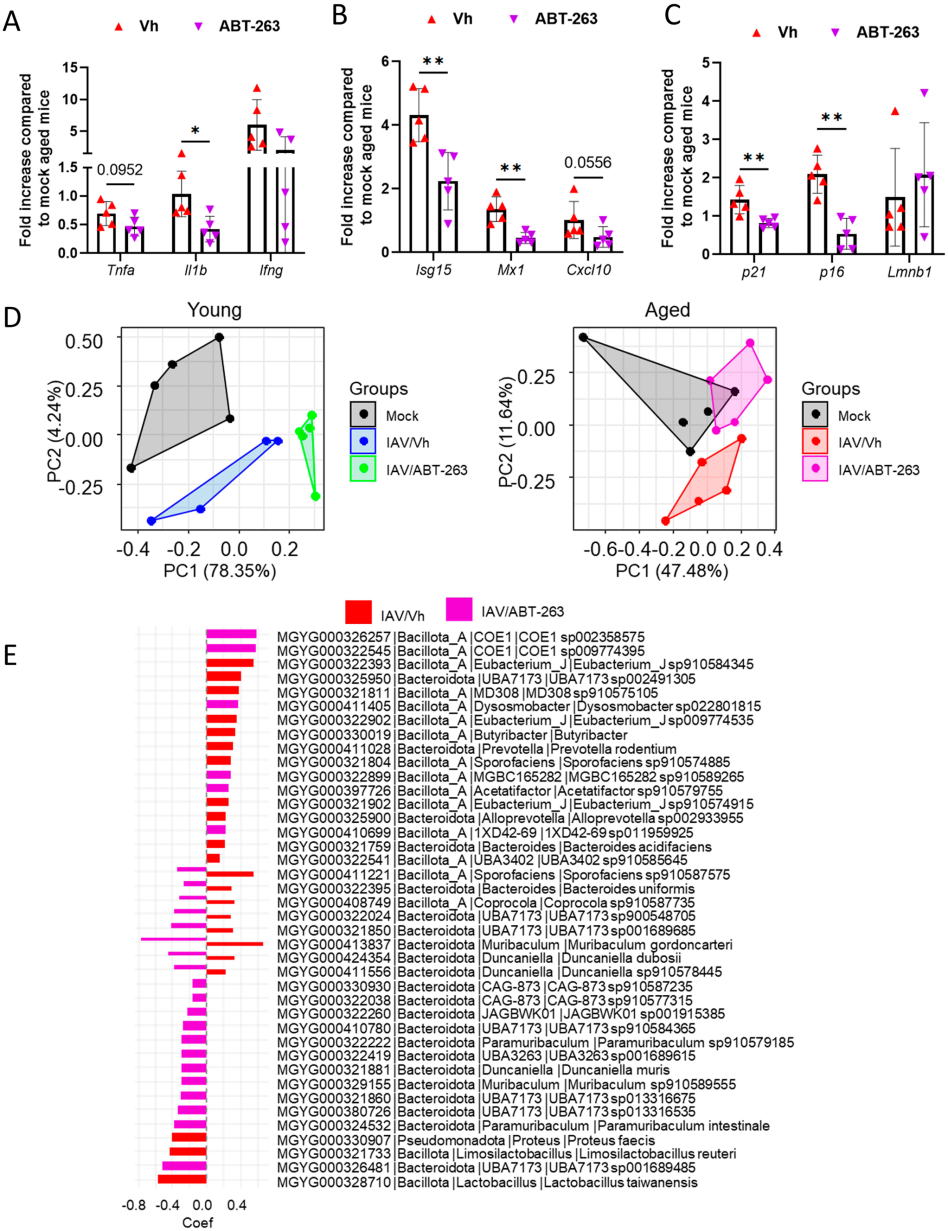
Effect of ABT‐263 treatment on gut disorders. Infected mice were sacrificed at 7 dpi. (A–C) mRNA copy numbers were quantified by quantitative RT‐PCR (jejunum, 7 dpi). The data are expressed as the mean of change relative to average gene expression in non‐infected aged animals. Errors indicate mean ± SD (*n* = 5). (D, E) Effects of ABT‐263 on IAV‐associated gut dysbiosis. (D) PCA score plots of MAG abundance quantified in mice feces. PCA was performed using the normalized and log2‐transformed intensities for gut microbiota MAGs. PCA score plots were generated with R ggplot2 with facet according to time points. (E) Significantly changed species in aged mice identified with MaAsLin2 with default parameters (Linear Model method was used for analysis, BH correction for calculating *q*‐values, and a *q*‐value threshold of 0.25). Differences between mock‐infected and IAV‐infected age mice are shown in red and differences between vehicle‐treated and ABT‐263‐treated infected mice are shown in violet. (A–C) One representative experiment out of two performed are depicted. Significant differences were determined using the Mann Whitney *U* test (*p < 0.05, **p < 0.01). (D, E) One experiment performed (*n* = 5).

Severe influenza can induce extrapulmonary disorders that affect disease outcomes (Froggatt and Heaton 2022), a phenomenon that is exacerbated with aging (Bartley et al. 2016). For example, via the lung‐gut axis, influenza promotes intestinal inflammation (Wang et al. 2014; Sencio, Gallerand, et al. 2021). To determine whether depletion of senescent cells affects intestinal inflammation, inflammatory cytokine levels were measured in intestinal tissues. Interestingly, ABT‐263 tended to reduce the levels of transcripts encoding TNF‐α, IL‐1β (significant), and IFNγ in aged intestine (Figure 3A). In parallel, it lowered the gene expression levels of *Isg15*, *Mx1*, and *Cxcl10* (Figure 3B). The effects exerted by ABT‐263 were associated with an altered expression of senescent markers such as *p21*, *p16* (diminished), and *Lmnb1* (slightly enhanced) in aged gut (Figure 3C). The effect of ABT‐263 was age‐specific with the exception of *Il1b* gene expression (Figure S3A–C).

We and others have demonstrated that influenza alters gut microbiota composition and function and that the resulting dysbiosis contributes to disease severity (Yildiz et al. 2018; Sencio et al. 2020; Heumel et al. 2024). The impact of aging on respiratory virus‐induced dysbiosis has recently been reported (Brito Rodrigues et al. 2025; Creskey et al. 2025; Bogard et al. 2025). We investigated whether age‐related senescent cells contribute to influenza‐associated gut dysbiosis. To this end, we analyzed the fecal gut microbiota composition using a metaproteomic approach (Creskey et al. 2025). As expected, principal component analysis (PCA) revealed obvious differences in the bacterial communities between mock‐infected and IAV‐infected groups in both young and aged mice (Figure S3D). Of interest, the composition of the gut microbiotas changed upon ABT‐263 treatment in both young and aged animals (Figure 3D). In aged mice, and relative to the uninfected group (mock), the microbial community of the ABT‐263‐treated group tended to return to basal levels. On the other hand, major differences were still observed in both vehicle‐treated and ABT‐263‐treated infected young animals. In aged mice, MaAsLin analysis identified six taxa as significantly up‐regulated and 20 taxa as significantly down‐regulated following ABT‐263 treatment (Figure 3E). Interestingly, some species found to be augmented after influenza had a decreased frequency after ABT‐263 treatment. This included *Sporofaciens Sp910587575*, *Bacteroides uniformis* , *Coprocola Sp910587735*, two *Bacteroideta* species (*sp900548705* and *sp001689685*), *Muribaculum gordoncarteri*, *Duncaniella dubosii*, and *Duncaniella Sp910578445*. Notably, the reduced frequencies of these bacterial species positively correlated with lower expression of inflammatory markers in both lungs and gut (Figure S3E). In young mice, ABT‐263 significantly altered microbial composition (162 taxa up‐regulated, 48 taxa down‐regulated), but not in a manner that reversed the changes induced by IAV infection (Figure S4). Together, our metaproteomics analysis suggests a role for age‐related cellular senescence on virus‐induced dysbiosis.

### 2.4 ABT‐263 Treatment Had No Effect on IAV‐Specific Antibodies but Alleviates CD8 T Cell Response in Aged Mice

**FIGURE 4:**
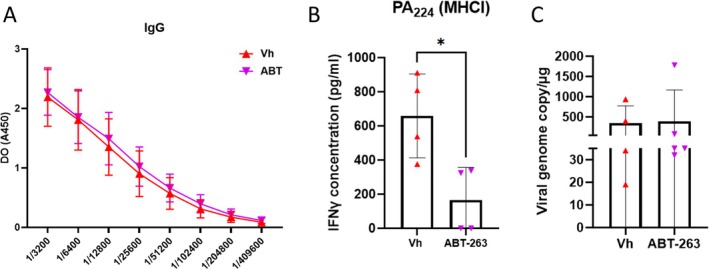
Effect of ABT‐263 treatment on IAV‐associated immune responses in aged mice. (A, B) IAV‐infected mice were sacrificed at 28 dpi. (A) Serum samples were collected and IgG titers were determined by indirect ELISA. (B) Spleen cells were restimulated with the MHC class I‐restricted peptide PA_224–233_ for 48 h. IFN‐γ production was assessed by ELISA. IFNγ production was not detected in mock‐infected mice. (C) Aged mice were re‐infected 28 days after the primary infection. Viral load in lungs was quantified 2 days after the challenge using specific TaqMan RT‐qPCR. Data are expressed as genome copy number (M1 protein)/μg RNA. One representative experiment out of two performed is depicted (*n* = 4–5). Significant differences were determined using the Mann Whitney *U* test (*p < 0.01).

Recent evidence indicates that age‐related senescent cells play a dual role in regulating immune responses (Cobanoglu et al. 2023), including in the context of influenza (Torrance and Haynes 2022). To investigate this further, we measured the generation of IAV‐specific antibodies at 28 dpi. ABT‐263 treatment did not significantly alter antibody production in either young (slightly augmented) or aged mice (Figures 4A and S5A, respectively). We then measured the antigen specific T cell response. ABT‐263 treatment reduced the production of IFN‐γ by aged splenocytes after restimulation with the MHC class I (H2b)‐restricted peptide PA_224–233_ (Figure 4B). This is in agreement with the observations of Torrance and collaborators (Torrance and Haynes 2022). In contrast, ABT‐263 augmented IFN‐γ production by young splenocytes (Figure S5B). We then investigated whether ABT‐263 treatment alters immune‐based protection against reinfection. To this end, mice were challenged with IAV (the same strain was used) 28 days after the primary infection. Viral loads were identical between vehicle‐treated and ABT‐263‐treated mice indicating that ABT‐263 treatment does not affect pulmonary defense against reinfection (Figure 4C and data not shown).

### 2.5 ABT‐263 Treatment Mitigated Pulmonary Sequelae in IAV‐Infected Aged Mice

**FIGURE 5:**
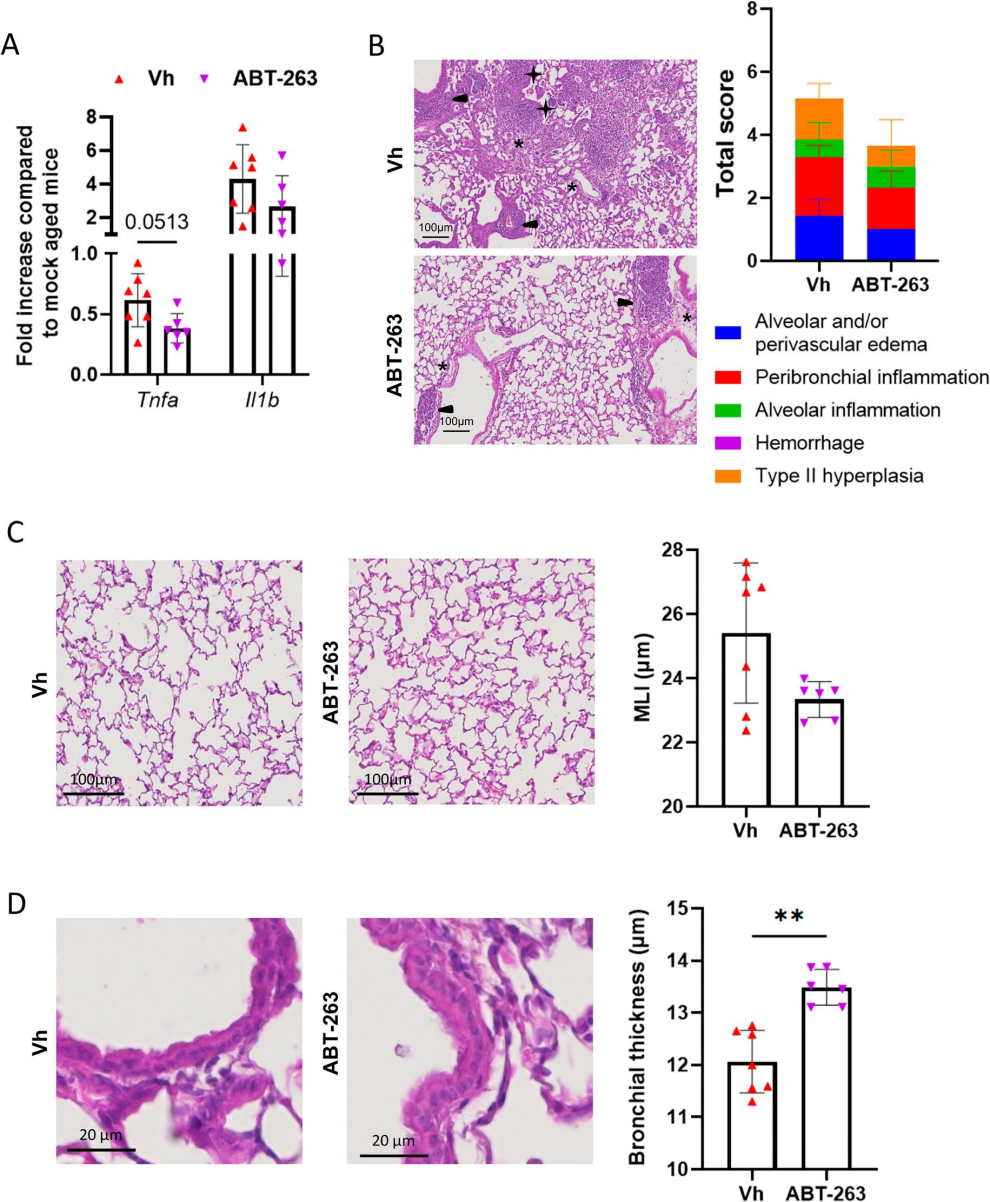
Effect of ABT‐263 treatment on long sequelae post‐influenza. IAV‐infected mice were sacrificed at 28 dpi. (A) mRNA copy numbers in jejunum were quantified by quantitative RT‐PCR. The data are expressed as the mean of change relative to average gene expression in non‐infected aged animals. (B) Left panels, Lungs were stained with H&E and histopathological examination of lung sections was performed. Right panel, The mean sum of the subscores is shown. (C) Measurement of emphysema. Left panels, Representative micrographs. *Right* panel, plot showing mean liner intercept (MLI) measurements. (D) Left panel, Representative micrographs showing airway epithelium. Right panel, Scatter‐plot graph showing bronchial wall thickness. One representative experiment out of two performed is depicted (*n* = 6–7). Significant differences were determined using the Mann Whitney *U* test (**p < 0.01).

Influenza infection can lead to long‐term pulmonary and extrapulmonary sequelae, particularly in aged individuals (Keilich et al. 2019; Xie et al. 2024). Preclinical models of aging have been shown to reproduce some of the persisting outcomes of influenza (Bartley et al. 2016; Goplen et al. 2020). We investigated whether early depletion of senescent cells in aged mice could modulate long‐term inflammation in the gut and lungs. In the gut, ABT‐263 tended to reduce the expression of transcripts encoding TNF‐α and IL‐1β at 28 dpi (Figure 5A). A histochemical analysis (H&E staining) of lung sections revealed pulmonary lesions which were higher in aged animals than in young counterparts (Figures 5B and S6A). Total histological scoring indicated a decrease of lung pathology in ABT‐263‐treated aged mice (*p* = 0,1055), but not in ABT‐263‐treated young mice. In particular, the alveolar and perivascular edema, inflammation and type II hyperplasia were mitigated (Figure 5B). Furthermore, lung emphysema lesions (assessed by measurement of the mean linear intercept) tended to be reduced (Figure 5C, *p* = 0.1375). We then measured the potential impact of early senescent cell depletion on fibrosis. The percentage of Sirius Red staining within alveolar walls was equivalent in vehicle‐treated and ABT‐263‐treated aged mice, indicating no change on collagen deposition (Figure S6B). In line, the levels of collagen 1, alpha 1 was not reduced after ABT‐263 treatment (Figure S6C). Lastly, we measured bronchial airway epithelium thickness. Interestingly, by 28 dpi, the airway epithelium completely recovered in ABT‐263‐treated aged mice (Figure 5D and not shown); this indicated marked acceleration of the epithelial repair process. In line with our prior findings (Lipskaia et al. 2025), ABT‐263 inoculation also accelerated the epithelial repair process in young animals (Figure S6D). Altogether, pharmacological depletion of senescent cells mitigated pulmonary sequelae in aged mice. With the exception of epithelial repair, these effects were age‐specific, suggesting that pre‐existing senescent cells were involved.

## 3 Discussion

Among mechanisms potentially involved in the severity of viral infections in aged individuals, cellular senescence might be a critical contributor (Camell et al. 2021; Delval et al. 2023; Torrance and Haynes 2022). The putative role of preexisting, age‐related senescent cells in influenza has recently been investigated. Two independent studies have reported that depletion of senescent cells (ABT‐263) or targeting the SASP factor prostaglandin E_2_ produced by age‐related pulmonary senescent cells reduces viral load, lowers lung inflammation and ameliorates mouse survival (Chen et al. 2022; Jiang et al. 2025). In these studies, age‐related senescent type II alveolar epithelial cells were suggested to be important. In a genetic model of aging, Lv and colleagues found that senolytic agents (fisetin and dasatinib/quercetin) reduced lung inflammation and pathology (but not the viral load), probably via reduction of aberrant activation of macrophages (Lv et al. 2022). Moreover, the genetic depletion of cells expressing the senescent marker p16 in aged mice enhanced viral clearance but reduced primary and memory immune responses (Torrance et al. 2024). On the other hand, two studies reported that senolytics, whether administered before or after infection, had no effect on viral load, lung damage, or the survival of aged mice (Torrance et al. 2023; Luna et al. 2025). In view of these discordant results, we revisited the potential effect of pharmacological senescent cell removal on influenza severity in an aged mouse model.

In line with the literature (Lv et al. 2022; Torrance et al. 2023; Luna et al. 2025), and at least at 7 dpi which is the peak of the inflammatory response in our experimental model, senolytic treatment had no effect on IAV replication in aged mice. This contrasts with the lower IAV replication due to genetic (selective) depletion of p16‐positive cells in aged mice reported by Torrance and colleagues (Torrance et al. 2024). The effect of senolysis in aged individuals on viral replication is likely to be context‐dependent (e.g., pharmacological vs. genetic depletion, spectrum of targeted cells) and virus‐dependent. In the case of SARS‐CoV‐2, elimination of senescent pulmonary epithelial cells, which express high levels of the SARS‐CoV‐2 receptor angiotensin‐converting enzyme 2 (Ma et al. 2022), results in lower viral load in infected aged individuals (Delval et al. 2023). Moreover, removal of senescent cells from aged brain organoids reduced viral load (Aguado et al. 2023). Along with enhanced virus entry, age‐related, pulmonary senescent cells might also favor viral replication (de Moraes et al. 2021). Whether IAV infects or replicates differently in age‐related senescent cells compared to younger cells remains to be determined for instance using in vitro cultures of cell‐sorted epithelial cells. IAV infection of pre‐existing senescent cells may amplify their proinflammatory SASP. In our setting, ABT‐263 reduced the intensity of the inflammatory response and lowered histopathological scores at 7 dpi. This beneficial effect might be due to the elimination of pre‐existing senescent cells as well as of senescent cells arising from direct viral infection and paracrine SASP (secondary senescence). Some lung and systemic SASP factors were reduced upon ABT‐263 inoculation such as IL‐6 and IFN‐γ. Interestingly, during severe influenza, IL‐6 production by the injured lung contributes to impaired muscle recovery, particularly in aged individuals (Radigan et al. 2019; Runyan et al. 2020). It remains to be determined whether reductions in IL‐6 levels following ABT‐263 treatment contribute to better enhanced muscle recovery after influenza infection. The reduction of lung inflammation on 7 dpi may have consequences at later time points. Indeed, ABT‐263 treatment (which was stopped at 7 dpi) alleviated some parameters associated with pulmonary sequelae at 28 dpi. It included edema, type II pneumocyte hyperplasia, emphysema, and airway epithelium damage both being known to infer with pulmonary functions. These findings are consistent with our previous results on COVID‐like disease (Delval et al. 2023). Elderly individuals are at increased risk of developing and dying from pneumonia. Moreover, elderly pneumonia survivors are at higher risk of developing age‐related disorders, including persistent lung dysfunction. Our findings are therefore important and suggest that specific depletion of pre‐existing senescent cells might ameliorate pulmonary outcomes following viral pneumonia. In the future, it will be interesting to study the consequences of senolysis not only on pulmonary dysfunction but also on extra‐pulmonary disorders, such as skeletal muscle atrophy (which limits mobility), heart and kidney damage, and cognitive impairment. It is noteworthy that the effects of ABT‐263 on pulmonary sequelae post‐viral pneumonia were age‐specific with little influence (epithelial damage) in young animals in line with Delval et al. (2023) and Lipskaia et al. (2025).

Regarding the importance of the gut‐lung axis in influenza severity (Sencio, Machado, et al. 2021), we investigated whether ABT‐263 could influence intestinal inflammation and gut dysbiosis resulting from IAV infection. This question is of interest given the pioneer study of Dandolini Saccon and colleagues demonstrating that senolytic (dasatinib/quercetin) treatment reduces intestinal senescence and inflammation while altering specific microbiota signatures in aged mice (Saccon et al. 2021). Our data clearly show that ABT‐263 treatment impacts the composition of the gut microbiota in IAV‐infected aged mice. Notably, ABT‐263 reduced the abundance of some taxa found to be up‐regulated by influenza. This included *Sporofaciens*, *Bacteroides*, *Bacteroideta*, *Muribaculum*, and *Duncaniella* members. Correlation analysis indicated that these species positively associated with markers of lung and gut inflammation. It is noteworthy that we observed age‐specific differences in the action of ABT‐263 in mice. Our study is the first to demonstrate that reducing cellular senescence through senolytic treatment mitigates the impact of acute viral pneumonia on gut dysbiosis. This finding is particularly important given the deleterious effects of IAV‐associated gut dysbiosis on lung inflammation (Heumel et al. 2024) and secondary bacterial infections (Sencio et al. 2020), which represent major causes of mortality in the elderly. Fecal transfer experiments will be necessary to demonstrate the potential impact of these changes on disease outcomes. Whether optimized senolytic regimens might improve influenza outcomes via reducing intestinal senescence, inflammation, and microbial dysbiosis in older subjects will be worth future studies.

Whether age‐associated senescent cells impair immune responses to antigenic challenge remains unclear (Torrance and Haynes 2022; Cobanoglu et al. 2023). We therefore investigated whether senescent cell removal in the context of influenza could modulate humoral and cellular immune responses in aged mice. Since the treatment regimen was initiated one day prior to infection and continued for 7 days, the effect of ABT‐263 was not limited to pre‐existing senescent cells but also influenced the ensuing antiviral immune response, either positively or negatively. We observed no effect on the humoral response indicating neither deleterious nor beneficial effects on antigen presentation and B cell development. In contrast, in line with Torrance and Haynes (2022) and Cobanoglu et al. (2023), the CD8 (memory) T cell response was strongly affected following ABT‐263 inoculation in aged mice. The effects were different in young animals (enhanced CD8 response), which indicates that the removal of (preexisting) senescent cells could have a causal role. Mechanisms are still unknown and worth of future studies. Regardless of the mechanism, ABT‐263 treatment did not exacerbate secondary influenza infection, underscoring the role of antibodies in resistance to reinfection.

Collectively, our findings suggest that pharmacologically clearance of senescent cells can partially reduce influenza severity in aged mice, primarily by alleviating acute and chronic inflammation in the gut and lungs. The present study has some limitations. We focused on an early senolytic intervention, targeting both pre‐existing senescent cells prior to infection (e.g., to reduce potential cellular targets for IAV and mitigate the SASP) and those arising during the early stages of infection. The effects of post‐infection ABT‐263 treatment (initiated at the onset of symptoms) in aged animals remain to be determined. Another limitation of the present study is its focus on ABT‐263, a drug that targets Bcl‐2 family proteins. The selective alleviation of cellular senescence should now be investigated using other classes of senolytics, including more targeted and less toxic second‐generation senolytics. Long term, intermittent senolytic administration is also envisaged. Lastly, genetic approaches, such as the selective depletion of p16‐ or p21‐expressing cells in aged mice, will also be critical to validate and extend our current findings. In conclusion, our results support previous evidence that pre‐existing cellular senescence is a potential risk factor for severe respiratory viral infections in the elderly (Chen et al. 2022; Lv et al. 2022; Delval et al. 2023; Jiang et al. 2025). However, to improve drug efficacy and minimize potential side effects, a deeper understanding of senescent cell properties—whether pre‐existing, virus‐induced, or SASP‐driven—will be necessary to optimize preventive and therapeutic strategies for mitigating the outcomes of viral pneumonia in the aged population.

## 4 Experimental Procedures

### 4.1 Animals and Ethics

Specific pathogen‐free C57BL/6J male mice were purchased from Janvier Labs (Le Genest‐St‐Isle, France). Young (2‐month‐old, 24–26 g) and aged (20‐month‐old, 38–42 g) mice were maintained in a biosafety level 2 facility at the Animal Resource Center of the Institut Pasteur de Lille for 2 weeks prior to experimentation for proper acclimatization. Mice had free access to standard rodent chow (SAFE A04, SAFE, Augy, France) and water. All experiments using mice complied with current national and institutional ethical guidelines and regulations (Institut Pasteur de Lille/B59‐350009). Protocols were approved by the regional Animal Experimentation Ethics Committee (Comité d'Ethique en Expérimentation Animale, Hauts de France, CEEA 75) and authorized by the French Ministry of Higher Education and Research (Ministère de l'Education Nationale, de l'Enseignement Supérieur et de la Recherche) (authorization number: APAFIS#27417–2020093019014739 v3).

### 4.2 Infection and Treatment With ABT‐263

For infection, mice were anesthetized via intraperitoneal administration of ketamine (1.25 mg) and xylazine (0.25 mg) diluted in 100 μL of phosphate buffered saline (PBS), before being intranasally infected with 50 μL of PBS containing 750 pfu of H1N1 A/California/04/2009 (pdm09) influenza virus. Control mice (mock‐infected) received 50 μL of PBS intranasally. To selectively eliminate senescent (Bcl2‐expressing) cells, mice were treated daily by oral gavage with ABT‐263 (Clinisciences, Nanterre, France) at a dose of 50 mg/kg, starting one day before infection and continuing until 7 days post‐infection (dpi). ABT‐263 was prepared at 10% in a vehicle composed of 30% polyethylene glycol 400 (#8.07485, Sigma‐Aldrich, Darmstadt, Germany) and 60% Phosal 50 PG (#NC0130871, Fisher Scientific, llkirch, France). Vehicle‐only treatments served as controls. Body weight and clinical scores (Table 1) were recorded daily, except beyond 18 dpi. The overall clinical score, ranging from 0 (normal) to 15, was calculated as the sum of individual parameter scores. Mice reaching a clinical score of 9 or higher were considered moribund and euthanized. Mice from both groups were sacrificed at defined time points post‐infection (7 dpi and 28 dpi). Fecal samples were collected at 7 dpi and stored at −20°C until DNA extraction. To study host defense against reinfection, mice previously infected with IAV were challenged with the same IAV strain at 28 dpi. Viral load was measured 2 days postinfection.

### 4.3 Quantification of Viral Load and Determination of Gene Expression by Quantitative RT‐PCR

Viral load and gene expression in the lungs were determined exactly as described (Lipskaia et al. 2025). Primer sequences are provided in Table 2. Gene expression data were normalized to *Gapdh* expression and presented as fold‐change relative to the mean gene expression levels in mock‐treated mice.

**TABLE 2:** Sequences of the oligonucleotides used in this study.

*Gapdh*	Forward 5′‐GCAAAGTGGAGATTGTTGCCA‐3′
	Reverse 5′‐GCCTTGACTGTGCCGTTGA‐3′
*Isg15*	Forward 5′‐GGCCACAGCAACATCTATGAGG‐3′
	Reverse 5′‐CTCGAAGCTCAGCCAGAACTG‐3′
*Mx1*	Forward 5′‐AGAAGGTGCGGCCCTGTATT‐3′
	Reverse 5′‐TGAACTCTGGTCCCCAATGACA‐3′
*Cxcl10*	Forward 5′‐ACCCAAGTGCTGCCGTCAT‐3′
	Reverse 5′‐CATTCTCACTGGCCCGTCAT‐3′
*Il6*	Forward 5′‐CAACCACGGCCTTCCCTACT‐3′
	Reverse 5′‐CCACGATTTCCCAGAGAACATG‐3′
*Ifng*	Forward 5′‐CAACAGCAAGGCGAAAAAG‐3′
	Reverse 5′‐GTGGACCACTCGGATGAGCT‐3′
*Tnfa*	Forward 5′‐CATCTTCTCAAAATTCGAGTGACA‐3′
	Reverse 5′‐TGGGAGTAGACAAGGTACAACCC‐3′
*Il1b*	Forward 5′‐TCGTGCTGTCGGACCCATA‐3′
	Reverse 5′‐GTCGTTGCTTGGTTCTCCTTGT‐3′

### 4.4 Lung Extracts, ELISA, and Western Blotting

Lung extracts were lysed in ice‐cold RIPA buffer (50 mM Tris–HCl, pH 7.4; 150 mM NaCl; 1 mM EDTA, 1% Triton X‐100) and 0.1% sodium deoxycholate supplemented with 1 mM PMSF and protease inhibitors (Roche Diagnostics, Basel, Switzerland). Lysates were centrifuged at 10,000 *g* for 5 min at 4°C to remove debris. Protein concentrations in the clarified supernatants were determined using the Pierce BCA Protein Assay kit (ThermoFisher Scientific). Samples were normalized to 1 mg/mL using the same lysis buffer. Cytokine levels were quantified by ELISA (R&D Systems). Western blotting was performed as described in Lipskaia et al. (2025). For detection of Col1a, a monoclonal rabbit antibody (1:1000, #72026S, Cell Signaling) was used, followed by incubation with a horseradish peroxidase‐conjugated secondary antibody (1:2500, #ab6789, Abcam). Protein bands were visualized using chemiluminescence (Pierce) and quantified using the “gel quantification” function in ImageJ software (version 1.1.0, NIH). For normalization, β‐actin was detected using a mouse monoclonal antibody (1:500, sc‐47778, Santa Cruz Biotechnology).

### 4.5 Histological Analysis, Immunohistochemistry and Immunofluorescence

Lung tissues (left lobe) were fixed in 4% PBS‐buffered formaldehyde for 48 h, rinsed in PBS, transferred into a 70% ethanol solution and processed into paraffin‐embedded tissue blocks. Histopathologic scores were given by a board‐certified pathologist. Tissue sections (3 μm thick) were stained with H&E reagent. Whole‐mount tissues were scanned with a Nanozoomer (Hamamatsu Photonics), and morphological changes were assessed by using a semi‐quantitative dual histopathology score including semi‐quantitative measurement of peribronchial and alveolar inflammation, bronchial lesions, edema, emphysema, hemorrhage, and type pneumocyte hyperplasia (scale from 0 to 4). To evaluate pulmonary fibrosis, the Sirius Red‐stained areas on scanned sections were measured with a computer‐assisted, automated, whole section histomorphometric image analysis technique (Visiopharm). Emphysema and epithelium thickness were measured as described (Lipskaia et al. 2025). Immunohistochemistry protocol was followed as detailed in the same reference. To quantify p21‐positive cells, entire lung sections were scanned using the Axioscan Z1 (Zeiss, Germany) and analyzed using QuPath open‐source software for bioimage analysis (https://qupath.github.io). Cells were classified as p21‐positive or negative based on the average intensity of nuclear DAB staining. p16 labeling and quantification of p16‐positive cells were performed as described (Lipskaia et al. 2025).

### 4.6 T Cell Restimulation and Antibody Detection by ELISA

Splenocytes (1 × 10^6^ cells) were restimulated for 48 h with 5 μg/mL of IAV‐specific class I MHC peptides (PA 224‐233: SSLENFRAYV, ProteoGenix, France). IFN‐γ levels in the culture supernatants were quantified by ELISA (#88‐7314‐88, ThermoFisher Scientific, Waltham, MA). For IAV‐specific antibody detection, 96‐well high‐binding plates (Dominique Dutscher, Bernolsheim, France) were coated with IAV antigen (ref), and 50 μL of samples diluted in PBS containing 0.5% (*w*/*v*) BSA were incubated at 37°C for 2 h. After washing, plates were incubated with an HRP‐conjugated anti‐mouse IgG antibody (1:2000) (*v*/*v*) in diluent solution (ThermoFisher Scientific) for 1 h at 37°C. The color reaction was developed by adding TMB substrate, and the enzymatic reaction was stopped with 100 μL of 2 N H_2_SO_4_. Absorbance was measured at 450 nm.

### 4.7 Fecal Protein Extraction, Trypsin Digestion and Desalting

Fecal samples were pre‐processed to enrich microbial cells using differential centrifugation, as described in Zhang et al. (2018). Briefly, fecal material was mixed with glass beads and cold PBS, vortexed, and centrifuged at 300× *g* at 4°C to collect the supernatant. Pellets underwent two additional PBS extractions, and the pooled supernatants were clarified by three sequential centrifugations at 300× *g*. Bacterial cells were then pelleted by centrifugation at 14,000× *g*, washed twice with PBS, and frozen for future analysis. Frozen microbial pellets were lysed in SDS/urea/TEAB buffer with ultrasonication. Lysates were centrifuged to remove debris, and proteins were precipitated using ice‐cold acetone, washed, air‐dried, and stored. For digestion, protein pellets were resuspended in urea/SDS buffer, quantified using a BCA assay, reduced with dithiothreitol, alkylated with iodoacetamide, and precipitated to remove SDS. Proteins were then reconstituted in urea/TEAB buffer and digested overnight with trypsin at a 1:25 enzyme‐to‐protein ratio. Digestion was stopped by adjusting the pH to 2–3 using formic acid. Peptides were desalted using Sep‐Pak C18 96‐well plate (Waters, Milford, USA) mounted on a vacuum manifold. Columns were conditioned with 50% acetonitrile (ACN) and equilibrated with 5% ACN/0.5% trifluoroacetic acid (TFA). Samples were loaded, washed with 5% ACN/0.5% TFA and 0.1% formic acid (FA), and eluted with 75% ACN/0.1% FA. Eluates were dried, resuspended in 0.1% FA, and diluted to 1 μg/μL for mass spectrometry (MS) analysis.

### 4.8 LC‐MSMS and Metaproteomic Data Analysis

Mass spectrometry (MS) analysis was performed using a timsTOF Pro 2 mass spectrometer (Bruker Daltonics, Bremen, Germany) coupled with a nanoElute 2 UPLC system (Bruker Daltonics, Bremen, Germany) operating in data‐independent acquisition mode (DIA‐PASEF), following using the protocols and settings previously described (Creskey et al. 2025). The resulted data independent analysis (DIA) data were processed with MetaLab_DIA for the identification, quantification, and taxonomic profiling of gut microbial proteins using the MGnify mouse gut metagenome‐assembled genome (MAG) database (v1.0). Metalab‐DIA was run in self‐model mode with an in‐house library derived from mouse gut microbial proteins. Taxonomic annotation was performed at the genome/MAG level with a scoring system incorporating the posterior error probability (PEP) and the cumulative intensity of all peptides assigned to a given genome. Genome information was obtained directly from the MGnify database. All MS proteomics data have been deposited to the ProteomeXchange Consortium (http://www.proteomexchange.org) under the dataset identifier PXD069493.

### 4.9 Statistical Analysis

Statistical analyses were performed using GraphPad Prism v9.0 (San Diego, CA) GraphPad Prism v8.0.2, and R software v4.0.2. Data for infectious markers are presented as mean ± standard deviation (SD), unless otherwise stated. Group comparisons were performed using the Mann–Whitney *U* test for two groups analyses, unless otherwise stated. For comparisons involving more than two groups, the nonparametric Kruskal–Wallis one‐way ANOVA was applied, followed by Dunn's post hoc test. Spearman correlation analysis was performed in R. Statistical significance was defined as *p < 0.05; **p < 0.01, ***p < 0.001.

## Author Contributions

François Trottein conceived and supervised the study. Lou Delval, Valentin Sencio, David Bernard, Serge Adnot, and François Trottein designed the experiments, and Lou Delval, Clara Valentin, Séverine Heumel, Lucie Deruyter, and Valentin Sencio performed the animal experiments. Lou Delval performed immunohistochemistry (p21 labeling), and Stefano Raviola and Larissa Lipskaia performed immunofluorescence (p16 labeling). Clément Bordas, Séverine Heumel, Lucie Deruyter, and Valentin Sencio managed the lung preparations and assessed pulmonary parameters. Lou Delval performed the western blotting; Clément Bordas, Séverine Heumel, and Lucie Deruyter performed the RT‐qPCR. Lucie Deruyter prepared feces samples, and Marybeth Creskey and Xu Zhang performed and analyzed the proteomic data. Isabelle Wolowczuk performed the correlation analysis. Philippe Gosset performed the histological scoring. Lou Delval designed the figures, and François Trottein drafted the manuscript. All the authors revised the manuscript and provided critical comments. François Trottein and Serge Adnot obtained fundings.

## Funding

This work was supported in part by the Institut National de la Santé et de la Recherche Médicale (Inserm), Centre National de la Recherche Scientifique (CNRS), University of Lille, Pasteur Institute of Lille. This work also benefited support from the French National Research Agency (Agence Nationale de la Recherche, ANR): ANR‐20‐CE14‐0023‐02 (INFLUENZAGING) (SA/FT/dB).

## Conflicts of Interest

The authors declare no conflicts of interest.

## Supporting information


**Figure S1:** Effect of ABT‐263 treatment on body weight and clinical scores. (A) Body weight loss and regain (A) and clinical scores (B) during the course of infection as represented by the area under the curve (AUC). Errors indicate mean ± SD (*n* = 5). One representative experiment out of two performed are depicted. Significant differences were determined using the Kruskal–Wallis test (**p < 0.01, ***p < 0.001).

**Figure S2:** Effect of ABT‐263 treatment on the acute phase response of influenza (young mice, 7 dpi). (A) Expression of p21 and Lmnb1 transcripts in the lung of IAV‐infected young mice as determined by RT‐qPCR. (B) Left panels, Representative photographs showing p21 expression in lung sections. Right panel, The percentages of p21‐positive cells are indicated. Scale bars, 100 μm. (C) Left panels, Lungs were stained with H&E. Representative photomicrographs are shown. Scale bars, 100 μm. Right panel, The mean sum of the subscores is shown. Errors indicate mean ± SD (*n* = 5). One representative experiment out of two performed are depicted. Significant differences were determined using the Mann Whitney *U* test (A and C) or the Kruskal–Wallis test (B) (* *p* < 0 0.05).

**Figure S3:** Effect of ABT‐263 treatment on gut disorders (young mice, 7 dpi). (A–C) mRNA copy numbers were quantified by quantitative RT‐PCR (jejunum). The data are expressed as the mean of change relative to average gene expression in mock‐infected young mice. (D) The gut microbiota compositions between mock‐infected and IAV‐infected young and old mice were compared. PCA score plots of MAG abundance quantified in mice feces. PCA was performed using the normalized and log2‐transformed intensities for gut microbiota MAGs. PCA score plots were generated with R ggplot2 with facet according to time points. (E) Associations between taxonomic and infectious features from IAV‐infected aged mice. Spearman correlation was used as similarity metric, with negative values represented in blue and positive values represented in red. (A–C) One representative experiment out of two performed are depicted. Errors indicate mean ± SD (*n* = 4–6). Significant differences were determined using the Mann Whitney *U* test (** *p* < 0.01). (D, E) One representative experiment out of two performed are depicted.

**Figure S4:** Effect of ABT‐263 treatment on IAV‐induced gut dysbiosis (young mice, 7 dpi). Significantly changed species identified with MaAsLin2 with default parameters (Linear Model method was used for analysis, BH correction for calculating *q*‐values, and a *q*‐value threshold of 0.25). Differences between mock‐infected and IAV‐infected young mice are shown in blue and differences between vehicle‐treated and ABT‐263‐treated infected mice are shown in green. One experiment performed (*n* = 5).

**Figure S5:** Effect of ABT‐263 treatment on IAV‐associated immune responses in young mice (28 dpi). (A) Serum samples were collected and IgG titers were determined by indirect ELISA. (B) Spleen cells were restimulated with the MHC class I‐restricted peptide PA_224–233_ for 48 h. IFN‐γ production was assessed by ELISA. IFNγ production was not detected in mock‐infected mice. One representative experiment out of two performed are depicted (*n* = 6–7). Significant differences were determined using the Mann Whitney *U* test (*p < 0.05).

**Figure S6:** Effect of ABT‐263 treatment on long sequelae post‐influenza (young mice, 28 dpi). (A) Lungs were stained with H&E and histopathological examination of lung sections were performed. Left panels, Representative photomicrographs are shown. Right panel, The mean sum of the subscores is shown. (B) Sirius Red labeling in the lungs of vehicle‐treated and ABT‐263‐treated young mice. The percentages of Sirius Red labeling are shown. (C) Expression of collagen 1, alpha 1 in vehicle‐treated and ABT‐263‐treated IAV‐infected, aged mice (whole lung homogenates). The relative protein levels normalized to β‐actine are shown (*n* = 5). (D) *Left* panels, Representative micrographs of H&E‐stained lung sections showing bronchial wall of IAV‐infected mice. *Right* panel, Scatter‐plot graph showing bronchial wall thickness. One representative experiment out of two performed are depicted (*n* = 4–5). Significant differences were determined using the Mann Whitney *U* test (*p < 0.05).

## Data Availability

The data that support the findings of this study are available from the corresponding author upon reasonable request.
