# Correction to “Senolytic Treatment Reduces Acute and Chronic Lung Inflammation in an Aged Mouse Model of Influenza”

**DOI:** 10.1111/acel.70586

**Published:** 2026-06-10

Delval, L., M. Creskey, C. Valentin, et al. (2026). “Senolytic Treatment Reduces Acute and Chronic Lung Inflammation in an Aged Mouse Model of Influenza.” *Aging Cell* 25, e70480. https://doi.org/10.1111/acel.70480.

In the published article, the author's name was misspelled as “Stephano Raviola.” The correct name spelling should be “Stefano Raviola.”

We apologize for this error.

