# Supplementary figures and images for: Senolytic Treatment Reduces Acute and Chronic Lung Inflammation in an Aged Mouse Model of Influenza
Source: Aging Cell. 2026 Apr 8;25(4):e70480. doi: 10.1111/acel.70480 (PMC13061596; doi:10.1111/acel.70480)

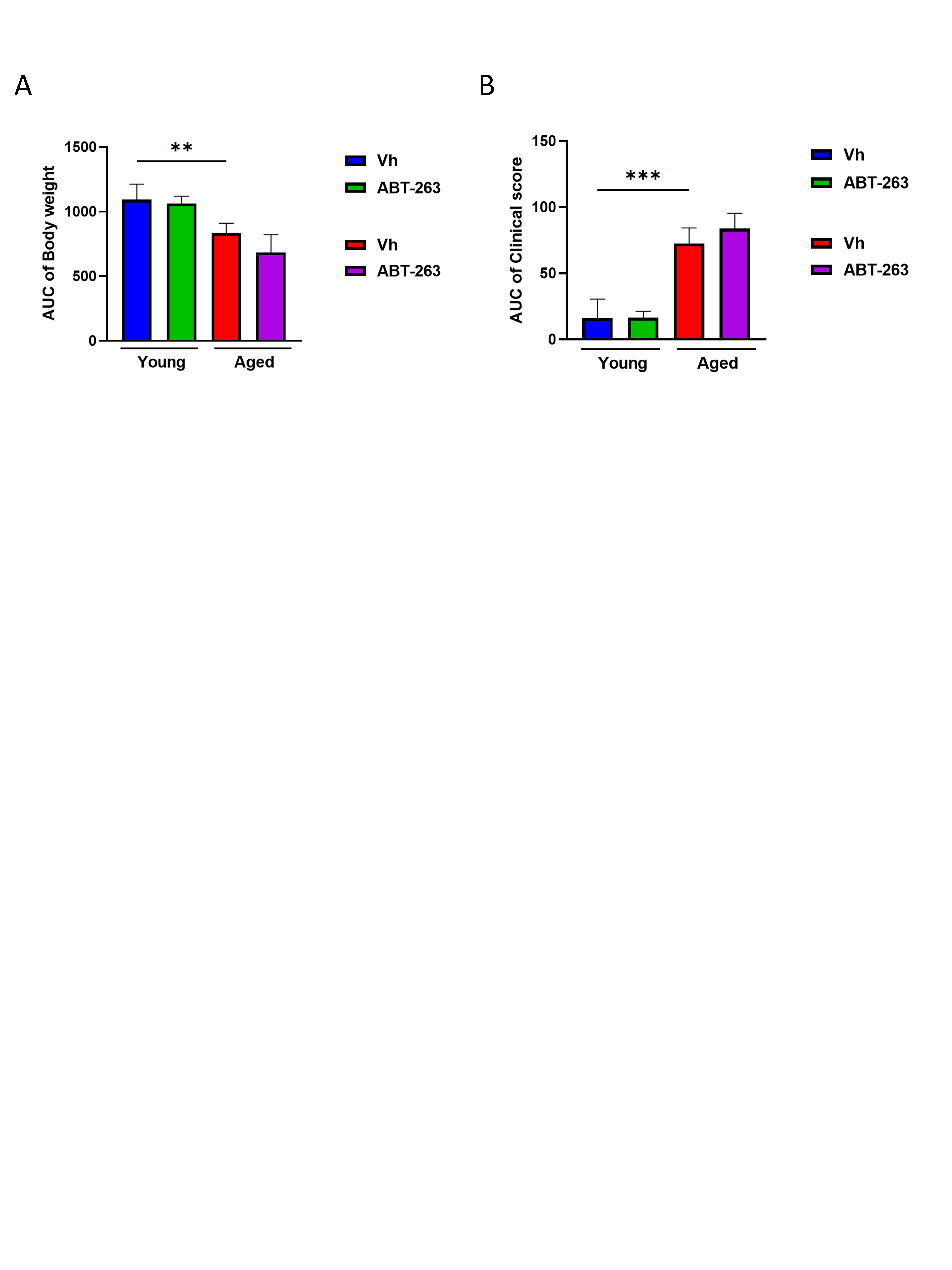

Supplement: Supplementary file 1 — Figure S1: Effect of ABT‐263 treatment on body weight and clinical scores. (A) Body weight loss and regain (A) and clinical scores (B) during the course of infection as represented by the area under the curve (AUC). Errors indicate mean ± SD (n = 5). One representative experiment out of two performed are depicted. Significant differences were determined using the Kruskal–Wallis test (**p < 0.01, ***p < 0.001). [file ACEL-25-e70480-s002.jpg]

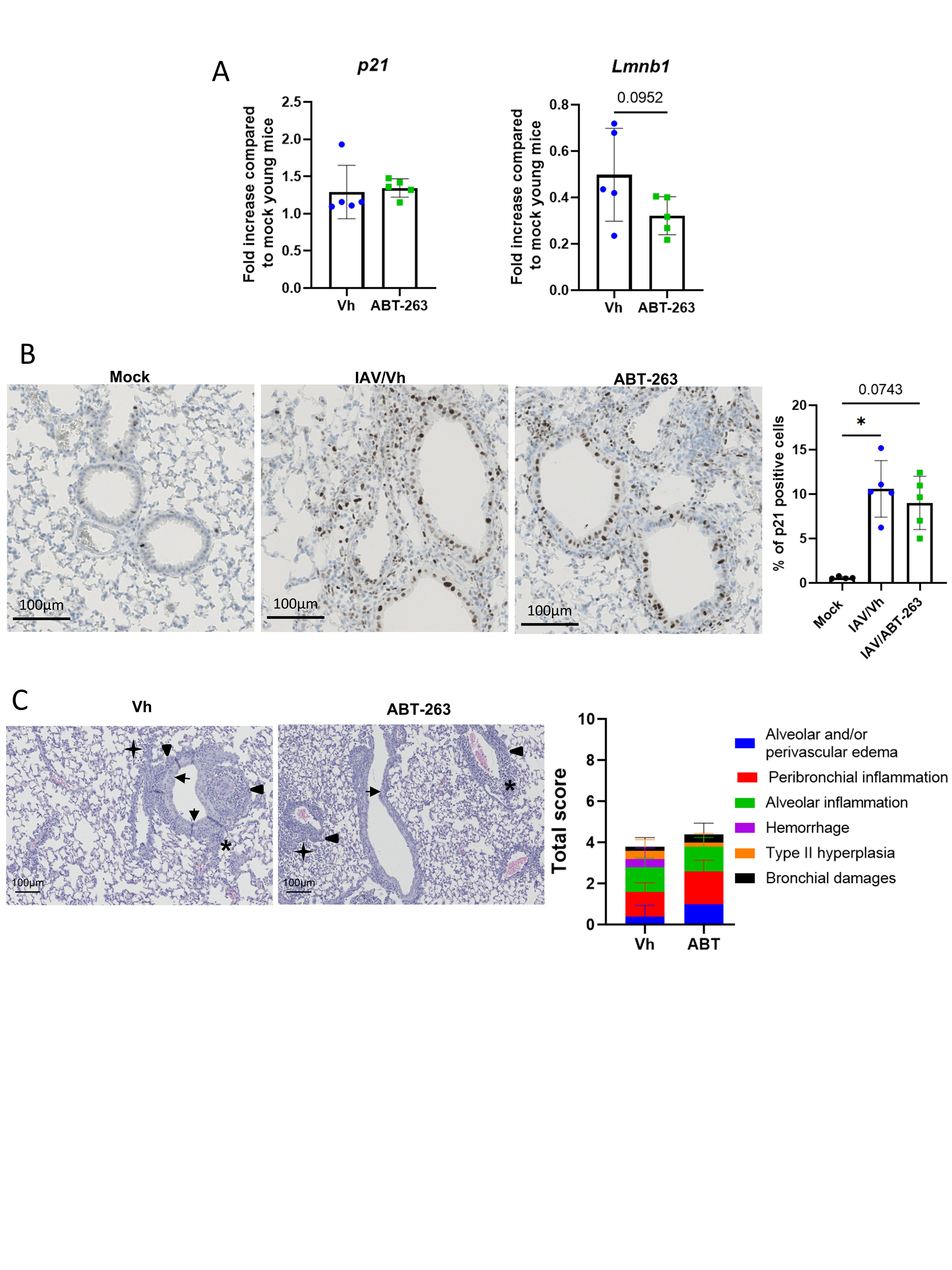

Supplement: Supplementary file 2 — Figure S2: Effect of ABT‐263 treatment on the acute phase response of influenza (young mice, 7 dpi). (A) Expression of p21 and Lmnb1 transcripts in the lung of IAV‐infected young mice as determined by RT‐qPCR. (B) Left panels, Representative photographs showing p21 expression in lung sections. Right panel, The percentages of p21‐positive cells are indicated. Scale bars, 100 μm. (C) Left panels, Lungs were stained with H&E. Representative photomicrographs are shown. Scale bars, 100 μm. Right panel, The mean sum of the subscores is shown. Errors indicate mean ± SD (n = 5). One representative experiment out of two performed are depicted. Significant differences were determined using the Mann Whitney U test (A and C) or the Kruskal–Wallis test (B) (* p < 0 0.05). [file ACEL-25-e70480-s004.jpg]

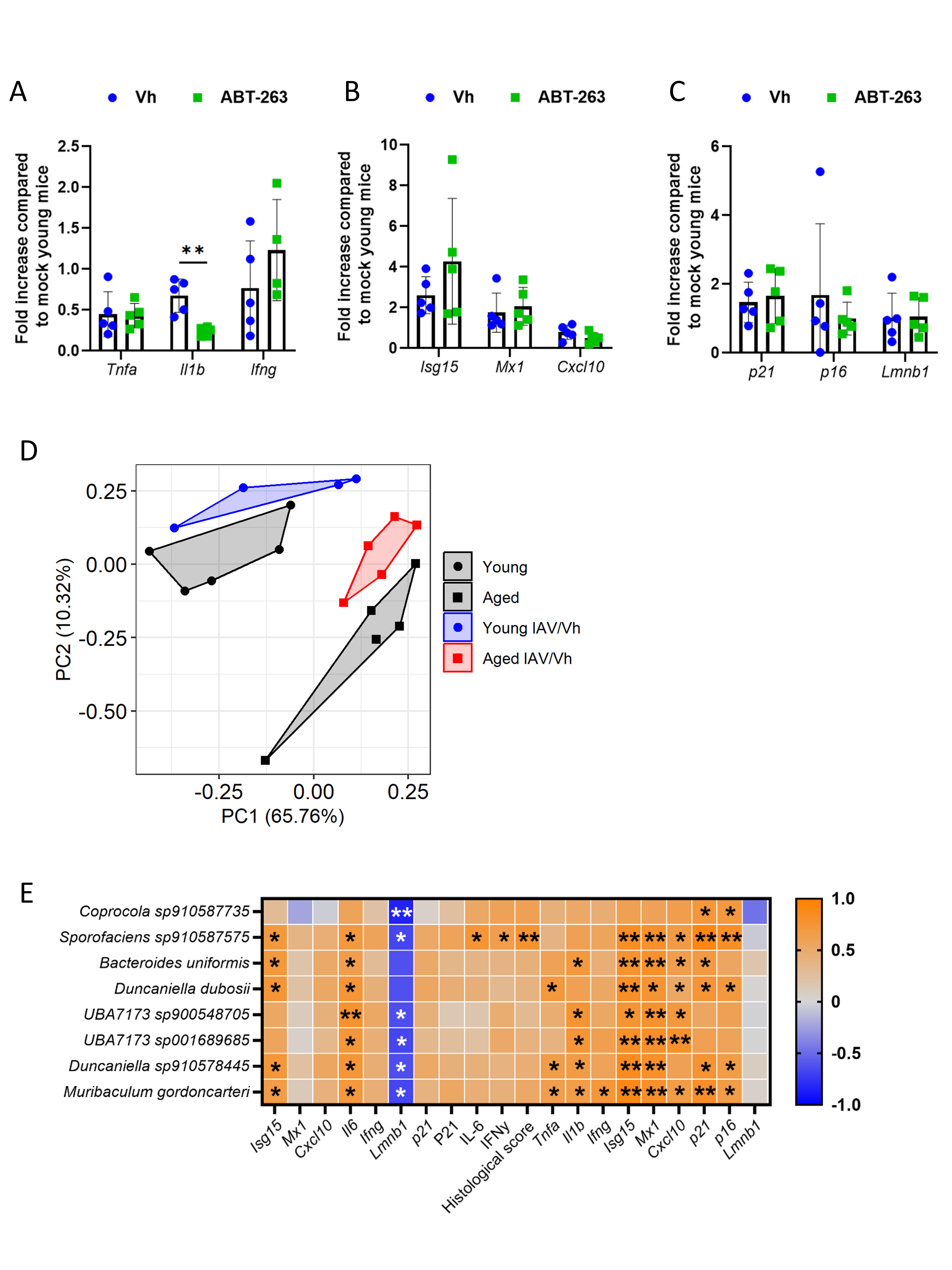

Supplement: Supplementary file 3 — Figure S3: Effect of ABT‐263 treatment on gut disorders (young mice, 7 dpi). (A–C) mRNA copy numbers were quantified by quantitative RT‐PCR (jejunum). The data are expressed as the mean of change relative to average gene expression in mock‐infected young mice. (D) The gut microbiota compositions between mock‐infected and IAV‐infected young and old mice were compared. PCA score plots of MAG abundance quantified in mice feces. PCA was performed using the normalized and log2‐transformed intensities for gut microbiota MAGs. PCA score plots were generated with R ggplot2 with facet according to time points. (E) Associations between taxonomic and infectious features from IAV‐infected aged mice. Spearman correlation was used as similarity metric, with negative values represented in blue and positive values represented in red. (A–C) One representative experiment out of two performed are depicted. Errors indicate mean ± SD (n = 4–6). Significant differences were determined using the Mann Whitney U test (** p < 0.01). (D, E) One representative experiment out of two performed are depicted. [file ACEL-25-e70480-s001.jpg]

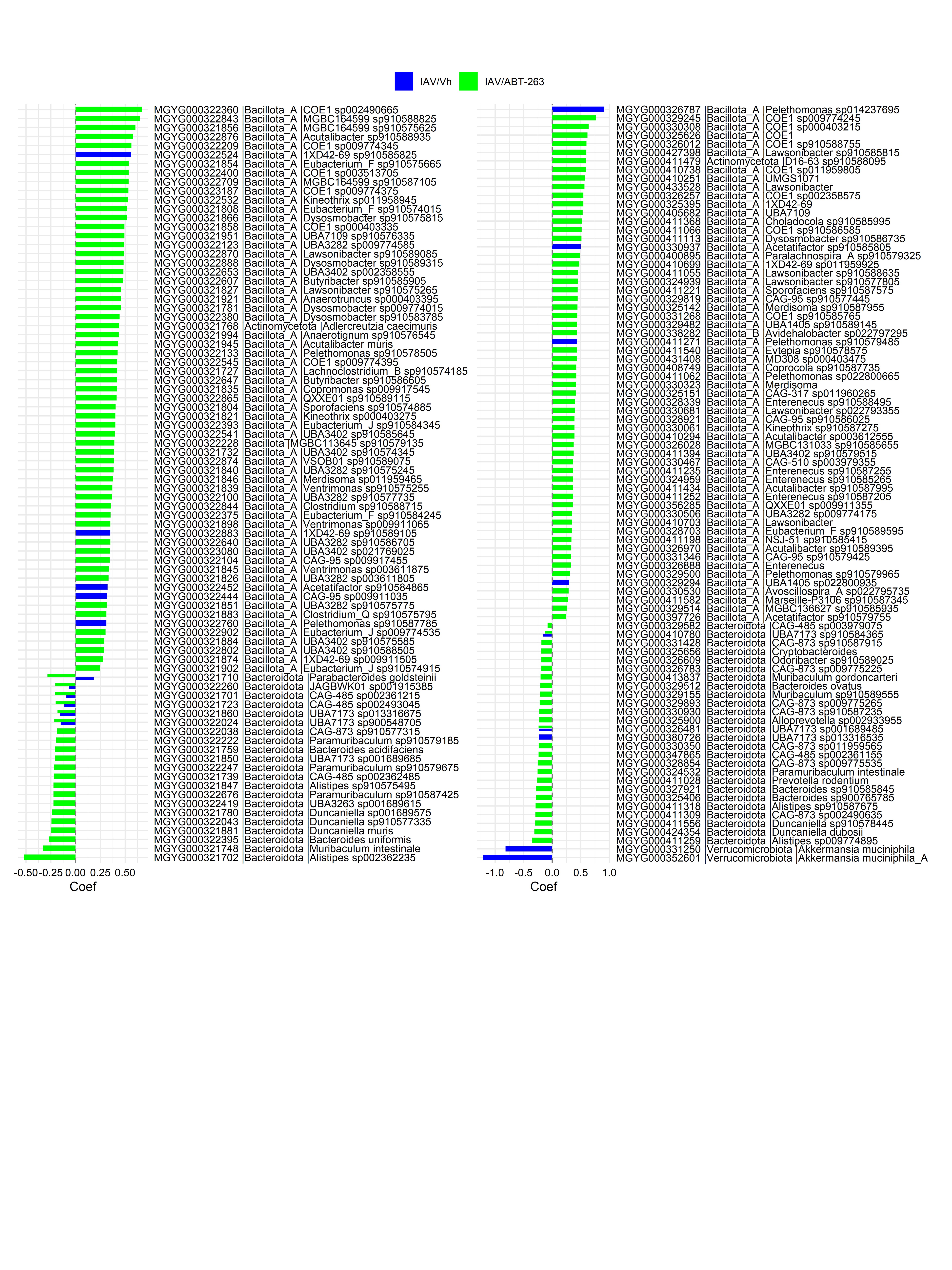

Supplement: Supplementary file 4 — Figure S4: Effect of ABT‐263 treatment on IAV‐induced gut dysbiosis (young mice, 7 dpi). Significantly changed species identified with MaAsLin2 with default parameters (Linear Model method was used for analysis, BH correction for calculating q‐values, and a q‐value threshold of 0.25). Differences between mock‐infected and IAV‐infected young mice are shown in blue and differences between vehicle‐treated and ABT‐263‐treated infected mice are shown in green. One experiment performed (n = 5). [file ACEL-25-e70480-s006.jpg]

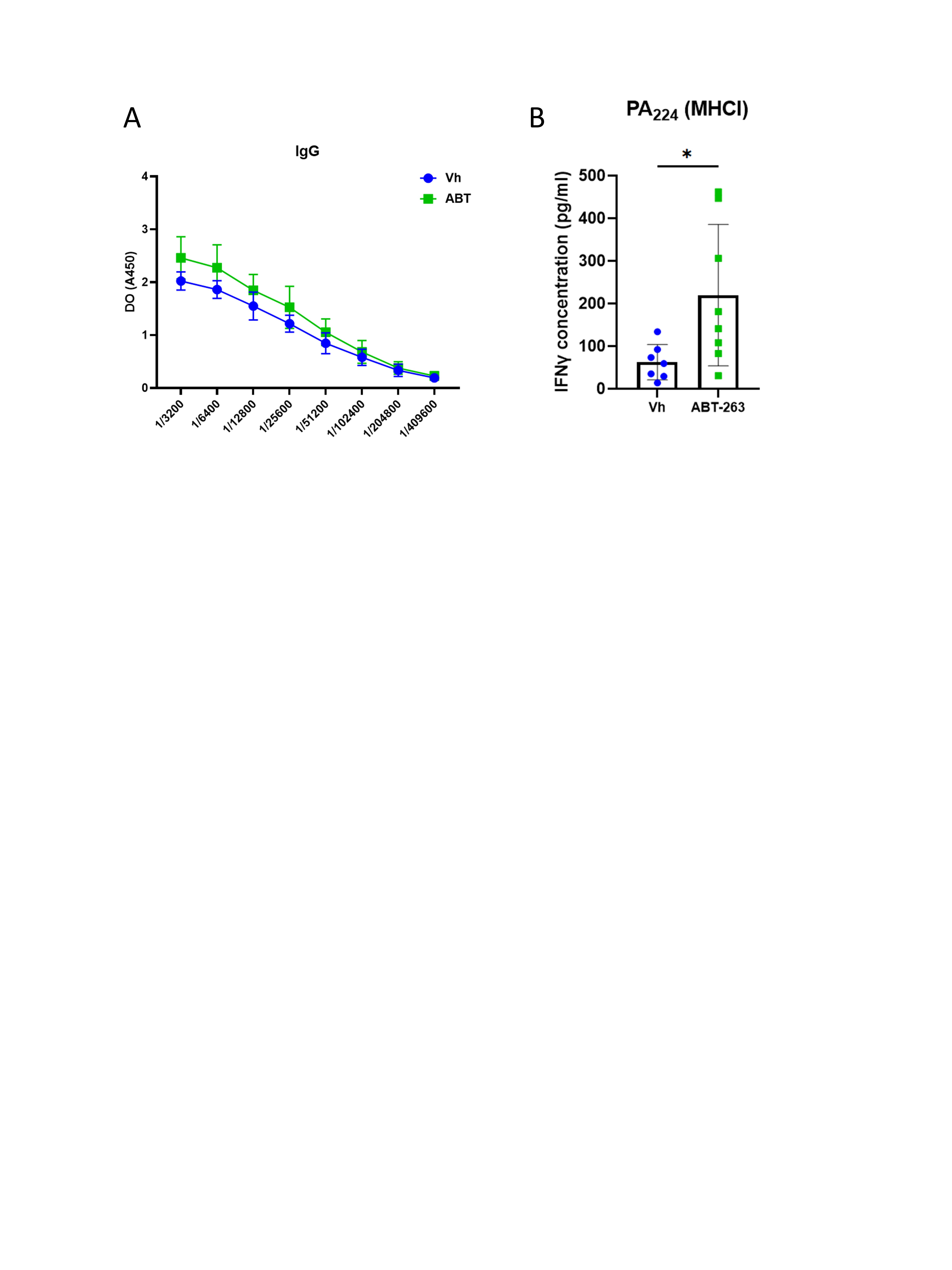

Supplement: Supplementary file 5 — Figure S5: Effect of ABT‐263 treatment on IAV‐associated immune responses in young mice (28 dpi). (A) Serum samples were collected and IgG titers were determined by indirect ELISA. (B) Spleen cells were restimulated with the MHC class I‐restricted peptide PA224–233 for 48 h. IFN‐γ production was assessed by ELISA. IFNγ production was not detected in mock‐infected mice. One representative experiment out of two performed are depicted (n = 6–7). Significant differences were determined using the Mann Whitney U test (*p < 0.05). [file ACEL-25-e70480-s005.jpg]

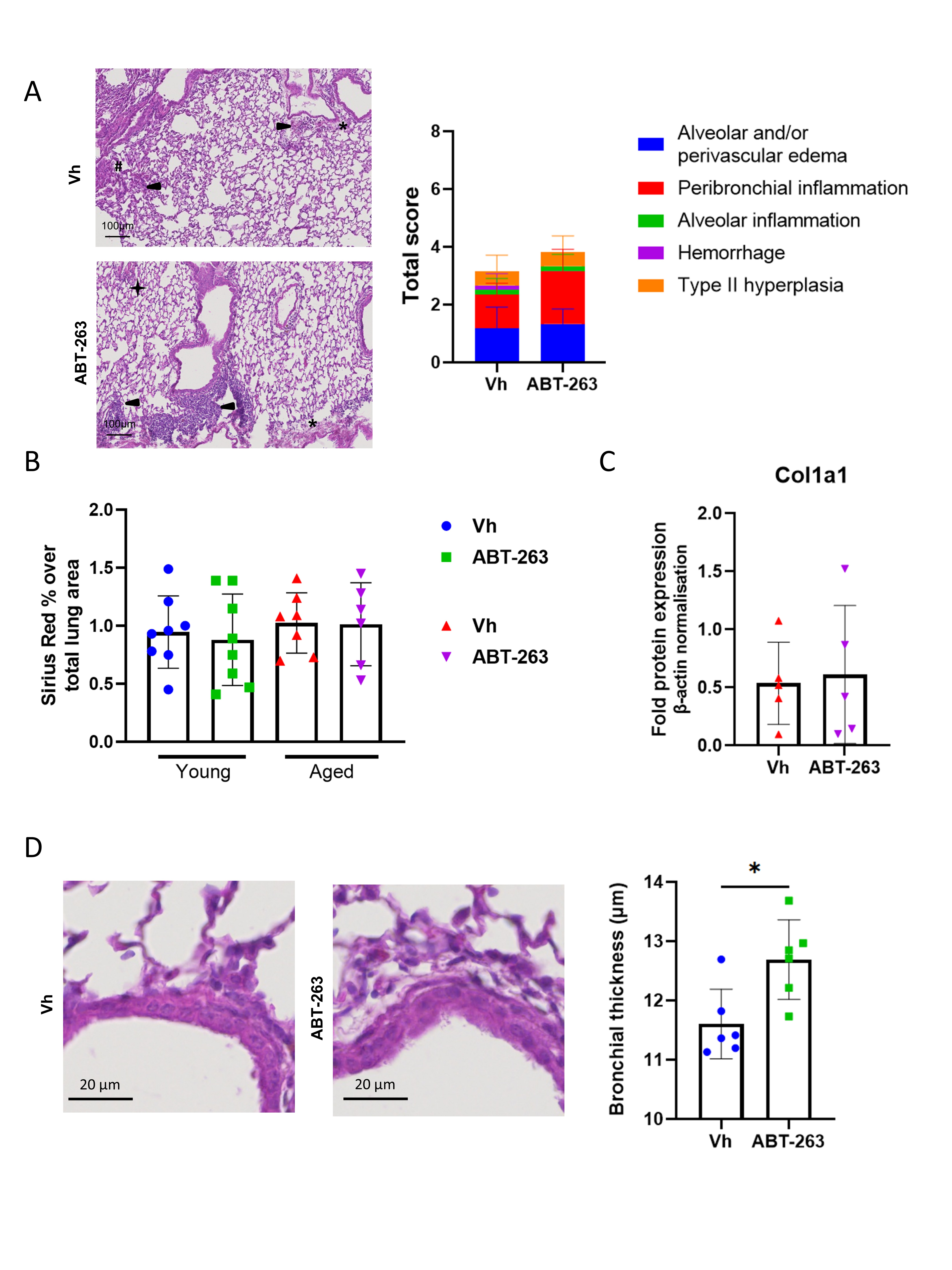

Supplement: Supplementary file 6 — Figure S6: Effect of ABT‐263 treatment on long sequelae post‐influenza (young mice, 28 dpi). (A) Lungs were stained with H&E and histopathological examination of lung sections were performed. Left panels, Representative photomicrographs are shown. Right panel, The mean sum of the subscores is shown. (B) Sirius Red labeling in the lungs of vehicle‐treated and ABT‐263‐treated young mice. The percentages of Sirius Red labeling are shown. (C) Expression of collagen 1, alpha 1 in vehicle‐treated and ABT‐263‐treated IAV‐infected, aged mice (whole lung homogenates). The relative protein levels normalized to β‐actine are shown (n = 5). (D) Left panels, Representative micrographs of H&E‐stained lung sections showing bronchial wall of IAV‐infected mice. Right panel, Scatter‐plot graph showing bronchial wall thickness. One representative experiment out of two performed are depicted (n = 4–5). Significant differences were determined using the Mann Whitney U test (*p < 0.05). [file ACEL-25-e70480-s003.jpg]
